# Apple Pomace Extract as a Sustainable Food Ingredient

**DOI:** 10.3390/antiox8060189

**Published:** 2019-06-21

**Authors:** Pedro A. R. Fernandes, Sónia S. Ferreira, Rita Bastos, Isabel Ferreira, Maria T. Cruz, António Pinto, Elisabete Coelho, Cláudia P. Passos, Manuel A. Coimbra, Susana M. Cardoso, Dulcineia F. Wessel

**Affiliations:** 1 QOPNA & LAQV-REQUIMTE, Department of Chemistry, University of Aveiro, 3810-193 Aveiro, Portugal; pedroantonio@ua.pt (P.A.R.F.); soniasferreira@ua.pt (S.S.F.); ritabastos@ua.pt (R.B.); ecoelho@ua.pt (E.C.); cpassos@ua.pt (C.P.P.); mac@ua.pt (M.A.C.); 2 Faculty of Pharmacy and Center of Pharmaceutical Studies, University of Coimbra, Azinhaga de Santa Comba, 3000-548 Coimbra, Portugal; isabelcvf@gmail.com (I.F.); trosete@ff.uc.pt (M.T.C.); 3 School of Agriculture and CI&DETS, Polytechnic Institute of Viseu, 3500-606 Viseu, Portugal; apinto@esav.ipv.pt; 4 CITAB, University of Trás-os-Montes e Alto Douro, 5001-801 Vila Real, Portugal; 5 CERNAS, Agrarian Higher School, 3045-601 Coimbra, Portugal

**Keywords:** polyphenols, antioxidant, anti-inflammatory, extraction, functional food

## Abstract

Apple pomace is a by-product of apple processing industries with low value and thus frequent disposal, although with valuable compounds. Acidified hot water extraction has been suggested as a clean, feasible, and easy approach for the recovery of polyphenols. This type of extraction allowed us to obtain 296 g of extract per kg of dry apple pomace, including 3.3 g of polyphenols and 281 g of carbohydrates. Ultrafiltration and solid-phase extraction using C18 cartridges of the hot water extract suggested that, in addition to the apple native polyphenols detected by ultra-high-pressure liquid chromatography coupled to a diode-array detector and mass spectrometry UHPLC-DAD-ESI-MS^n^, polyphenols could also be present as complexes with carbohydrates. For the water-soluble polyphenols, antioxidant and anti-inflammatory effects were observed by inhibiting chemically generated hydroxyl radicals (OH•) and nitrogen monoxide radicals (NO•) produced in lipopolysaccharide-stimulated macrophages. The water-soluble polyphenols, when incorporated into yogurt formulations, were not affected by fermentation and improved the antioxidant properties of the final product. This in vitro research paves the way for agro-food industries to achieve more diversified and sustainable solutions towards their main by-products.

## 1. Introduction

The fact that agro-food industrial by-products are generally disposed, often with great expenses and environmental implications, has raised the need for their valuation [1]. Their perishable nature, due to the high-water content and huge amounts of organic load, as well as their chemical composition, particularly in dietary fiber and phytochemicals, provides a costless source of bioactive compounds that may favor an efficient and sustainable industrial development [1,2]. As a matter of fact, a circular economy model can be implemented in the agro-food sector by recycling its by-products, thereby creating added value with fewer resources.

Among the wide variety of agro-food industrial by-products available worldwide, apple pomace, resultant from apple (*Malus* spp., Rosaceae) processing, can be highlighted given the ubiquitous presence of the fruit in the diet of all cultures [3]. Actually, every year millions of tons of apples are processed to produce apple cider, juices, or concentrates, which yield huge amounts of residues, comprising the pulp, skin, seeds, and stalks from the fruit [2]. Several strategies for the valuation of apple pomace have been proposed, including its direct use for animal feed, organic acids, enzymes, bioethanol and biogas production by microbial fermentation, or the development of new materials as part of biocomposites [4]. Nevertheless, this by-product still presents a significant edible fraction which can be used as a source of valuable components. These may include hydroxycinnamic acids (chlorogenic acid and *p*-coumaroylquinic acid), flavan-3-ols (monomers such as epicatechin to large polymers known as procyanidins), flavonols (quercetin rutinoside, galactoside, glucoside, xyloside, arabinoside, and rhamnoside derivatives), dihydrochalcones (phloretin 2-*O*-glucoside and phloretin 2-*O*-xyloglucoside), and anthocyanins (cyanidin 3-*O*-galactoside) [2,5,6]. Furthermore, given the occurrence of polyphenol oxidation reactions, polyphenols might also be found as components attached to cell wall polysaccharides [7,8], which may have an impact on the antioxidant and antiviral properties attributed to apple pomace extracts [9]. Most studies aiming to evaluate the potential applications of apple pomace valuable components have been performed with the use of organic solvents, which may be appropriate for pharmaceutical or cosmetic purposes but not for food applications.

Therefore, this work aims to give new insights into the nature of the apple pomace water-soluble polyphenols and their bioactivity, as well as to evaluate the potential of its water extract to be used for the supplementation or development of fortified products. To this end, in addition to apple native polyphenols, the occurrence of polyphenol/carbohydrate complexes was inferred employing ultrafiltration and solid-phase extraction of the hot water extract. Furthermore, the polyphenols isolated by solid-phase extraction were also used to provide evidence of their antioxidant and anti-inflammatory properties using both chemical and cellular inflammatory models. The feasibility of incorporating the aqueous extract of apple pomace into foods was tested by its addition to yogurt formulations and its potential impact on the fermentation process (pH, titratable acidity, and lactic acid counting), and antioxidant and nutritional properties of the final product.

## 2. Materials and Methods

### 2.1. Chemicals

All reagents were of analytical grade. All standard compounds used for polyphenol quantification by UHPLC-DAD-ESI-MS^n^ or antioxidant assays had a purity level of at least 95%.

### 2.2. Preparation of Extracts

**Figure 1:**
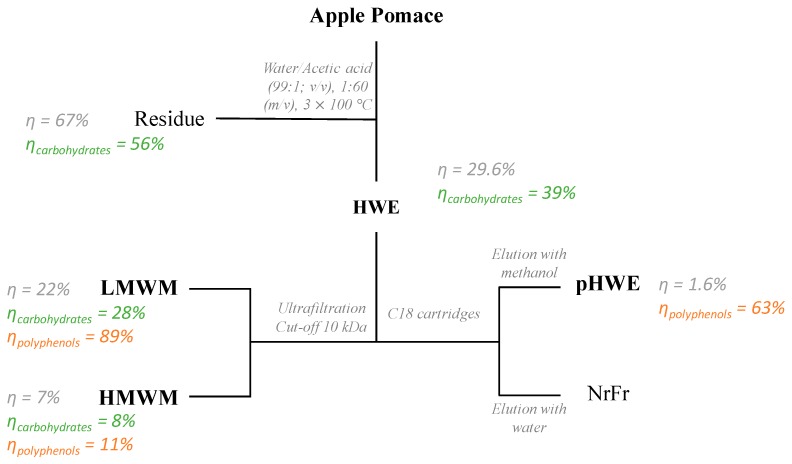
Schematic representation of the fractionation processes adopted in this work and yields of the extracts and polysaccharides in relation to apple pomace and polyphenols present in the hot water extract (dry basis). In bold are highlighted the fractions that were further studied. HWE—hot water extract; LMWM—low molecular weight material; HMWM—high molecular weight material; pHWE—purified hot water extract; NrFr—non-retained fraction.

Agro-industrial apple pomace was obtained following the general procedure described by Kennedy et al. [10]. This process consisted of the processing of a mixture of apples, mainly composed of the Royal Gala variety, employing milling, enzymatic digestion (amylase, pectin lyase, and polygalacturonase), and pressing processes for a period of at least 3 hours. After processing, the apple pomace was frozen at −20 °C, freeze-dried, sealed in bags, and stored in a dark at room temperature in a desiccator until further analysis. Extracts were prepared from apple pomace, using boiling water with 1% acetic acid, pH 2.5, at a solid (dry weight) to a solvent ratio of 1:60 (g/mL), the optimal conditions for polyphenol extraction as determined by Çam and Aaby [5]. The procedure was limited to a period of 10 min as the extraction yields (in mass) hardly improved for more than 10% using longer periods, e.g., 1 or 2 h, and allowed us to avoid the polyphenols thermal degradation [11,12]. Afterwards, the extracts were filtered (Whatman filter paper nº 4 and G3 sintered funnel), and the residue was re-extracted two more times following the same procedure to recover any remnant material. The crude extracts were combined, concentrated under reduced pressure, and freeze-dried, yielding a hot water extract (HWE). For characterization of the high and low molecular weight material (Figure 1), the HWE was fractionated at room temperature on an ultrafiltration module – Labscale TFF System (Merck KGaA, Darmstadt, Germany), using a pellicon XL ultrafiltration ultracel membrane with cut-off 10 kDa, as previously described by Passos et al. [13].

To further characterize the polyphenolic composition and potential bioactive effects, the HWE was submitted to solid-phase extraction (Figure 1) in Sep-Pak C18 cartridges (SPE-C18, Supelco-Discovery (St. Louis, MO, USA, 20 g). The column was preconditioned with 20 mL of methanol followed by 20 mL of water. Afterwards, the sample was loaded onto the column, and the non-retained material (NrFr) was eluted with water, three times the volume of the cartridge. The material retained was eluted using methanol following the same procedure. The resultant polyphenol-isolated HWE fraction (pHWE) was concentrated under reduced pressure to remove the methanol and then frozen and freeze-dried.

For the fractionation processes by ultrafiltration or solid-phase extraction using C18 cartridges, polyphenols yields were estimated by mass balance between those initially found in the hot water extract and those obtained in the further sub-fractions. For ultrafiltration it was estimated as described in Equation (1):

PCHWE = PCHMWM + PCLMWM(1)

where, PCHWE, PCHMWM, and PCLMWM corresponds to the mass of polyphenols present in the HWE, high molecular weight material fraction, and low molecular weight material fractions, respectively.

For solid phase extraction the yields were estimated considering Equation (2):

PCHWE = PCpHWE + PCNrFr(2)

where PCHWE, PCpHWE, and PCNrFr corresponds to the mass of polyphenols present in the HWE, in the fraction retained in the C18 cartridges (pHWE), and in the non-retained fraction in the C18 cartridge (NrFr), respectively. For polysaccharides, a similar rationale was applied taking as a reference the number of polysaccharides initially present in the apple pomace.

### 2.3. General Chemical Characterization

The moisture content of apple pomace was determined by the weight difference before and after drying for 12 h at 105 °C, up to constant weight. For apple pomace and derived extracts, protein was estimated by determining total nitrogen using a Truspec 630-200-200 elemental analyzer (St. Joseph, MI, USA) with a thermal conductivity detector (TDC) and employing a conversion factor of 5.72, as estimated for apples [14]. To obtain quantitative and qualitative information, the carbohydrate composition of the samples was determined by adopting the general procedure of Fernandes et al. [8]. Briefly, neutral sugars were determined using gas chromatography (GC) analysis after acid hydrolysis (12 M H_2_SO_4_ for 3 h at room temperature, followed by 2.5 h hydrolysis in 1 M H_2_SO_4_ at 100 °C), reduction with NaBH_4_ (15% w/v in 3 M NH_3_ during 1 h at 30 °C), and acetylation (with acetic anhydride in the presence of 1-methylimidazole during 30 min at 30 °C). Uronic acids were quantified by the 3-phenylphenol colorimetric method after acid hydrolysis (1 h in 1 M H_2_SO_4_ at 100 °C) of the sample [15]. Galacturonic acid (GalA) was used as standard. Free sugars were determined following the same procedure without the hydrolysis step. Fructose was quantified as the sum of mannitol and sorbitol due to the epimerization of fructose during the reduction step, using the ratio of the epimerization reaction [16]. The amount of total polyphenolic compounds was quantified using the Folin–Ciocalteu method [17] using concentrations from 1 to 10 mg of extract/mL water. The results were expressed as g gallic acid equivalent (GAE)/kg of extract.

Individual polyphenols were determined by UHPLC-DAD-ESI-MS^n^ on an Ultimate 3000 (Dionex Co., San Jose, CA, USA) apparatus equipped with a Diode Array Detector (Dionex Co., USA) and coupled to a mass spectrometer. The chromatographic system consisted of a quaternary pump, an autosampler, a degasser, a photodiode-array detector, and an automatic thermostatic column compartment. Hypersil Gold (Thermo Scientific, San Jose, CA, USA) C18 column (100 mm length; 2.1 mm i.d.; 1.9 μm particle diameter, end-capped) at 30 °C was used. The mobile phase was composed of (A) 0.1% (v/v) formic acid and (B) acetonitrile. The solvent gradient started with 5% of solvent (B), reaching 40% at 14 min and 100% at 16 min, followed by the return to the initial conditions. The flow rate was 0.1 mL min^−1^ and UV-Vis spectral data for all peaks were accumulated in the range 200–500 nm, while the chromatographic profiles were recorded at 280, 320, and 340 nm for polyphenol analysis. The mass spectrometer used was a Thermo LTQ XL (Thermo Scientific, USA) ion trap MS, equipped with an electrospray ionization (ESI) source. Control and data acquisition were carried out with the Thermo Xcalibur Qual Browser data system (Thermo Scientific, USA). Nitrogen above 99% purity was used, and the gas pressure was 520 kPa (75 psi). The instrument was operated in negative-ion mode with ESI needle voltage set at 5.00 kV and an ESI capillary temperature of 275 °C. The full scan covered the mass range from *m*/*z* 100 to 2000. CID–MS/MS and MS^n^ experiments were acquired for precursor ions using helium as the collision gas with an energy of 25–35 arbitrary units.

For quantitative analysis, calibration curves were performed by injection of 5 known concentrations of standard compounds. Detection (LOD) and quantification (LOQ) limits were calculated using the parameters of the calibration curves, defined as 3.3 and 10 times the value of the regression error divided by the slope, respectively.

For rutin (ACROS), the tested range was 1.0–10.0 µg/mL, and the equation was y = 12,624x − 953, with an *R*^2^ value of 0.999. The quantification limit (LQ) and detection limit (LD) for this compound were 1.29 and 0.43 µg/mL, respectively. For quercetin-3-*O*-glucoside (Sigma-Aldrich), the tested range was 2.4–12.2 µg/mL, the equation was y = 16,421x − 879 with an *R*^2^ value of 0.999. The LQ and LD were 1.07 and 0.35 µg/mL, respectively. The calibration curve of phloridzin (Sigma-Aldrich), y = 20,429x − 456, were performed for ranges of 2.3–11.7 µg/mL, presenting an *R*^2^ of 0.999. The determined LQ and LD were 1.35 and 0.45 µg/mL, respectively. The remaining quercetin derivatives were quantified as quercetin-3-*O*-glucoside equivalents.

### 2.4. Formulation of a Hot Water Extract (HWE)-Fortified Yogurt

The apple pomace HWE was used as an ingredient for yogurt formulations. Yogurts were prepared from ultra-high temperature pasteurized milk (composed of 5.1% of carbohydrates, 3.4% of protein, and 1.6% of fat) and milk powder at 1% w/w of milk (composed of 54% of carbohydrates, 34.5% of protein, and 1% of fat) in the absence of, or alternatively with the addition of, extract, to yield a control yogurt and a supplemented yogurt, respectively. A ratio of 3.3% (w of extract/w of milk) was used based on the maximum amount of extract soluble in milk after heating to 90 °C for 2 min and leaving to cool to 40 °C. Plain yogurt (composed of 4.0% of carbohydrates, 3.2% of protein, and 2.9% of fat), purchased at the local market, was added (1% w/w of milk) as inoculum to achieve a final Lactic Acid Bacteria count of 6 Log colony forming units (CFU)/g of mixture. This amount was determined based on the *Streptococcus thermophilus* counts present in the commercial yogurt and detected in M17 (Liofilchem, Rosetodegli Abruzzi, Italy), a medium specific for the growth of this bacterium. An incubation period of 72 h at 37 °C was used for *Streptococcus thermophilus* counting. For yogurt production, the mixture was incubated at 42 °C until reaching a pH below 4.5. The fermentation process was controlled by measuring the pH, titratable acidity (g of lactic acid/100 g), and *Streptococcus thermophilus* counts every 2 hours. For titratable acidity, samples were homogenized in water at a proportion of 1:9 (w/v). Afterwards, the pH value of the samples was measured, and titrated with 0.1 M NaOH in the presence of a few drops of phenolphthalein (1%) as an indicator. The titratable acidity was expressed in g of lactic equivalents/100 g of yogurt.

### 2.5. Antioxidant Activity

HWE and pHWE antioxidant activity was screened by the 2,2-diphenyl-1-picrylhydrazyl (DPPH•) [18] and 2,2′-azinobis-(3-ethylbenzothiazoline-6-sulfonic acid) (ABTS•^+^) methods [19], using water as a solvent. The results were expressed as half maximum effective concentration (EC_50_) (µg/mL), which represents the amount of extract required to reduce the radical concentration to half of its initial concentration. In addition, pHWE was evaluated for its capability to inhibit OH• radicals generated by the ferric-ascorbate-EDTA-H_2_O_2_ Fenton system, following the general procedure of Kunchandy and Rao [20]. The scavenging of OH• was measured by determining the relative amount of oxidized deoxyribose formed in the presence and absence of the extract. The results were expressed as mannitol equivalents (mmol/g of extract). As the OH• scavenging is based on the inhibition of deoxyribose oxidation by antioxidants, other sugars present, such as those found in the HWE, could interfere. For this reason, the antioxidant activity was measured on pHWE, obtained by purification of the HWE by solid-phase extraction.

To evaluate the effect of HWE addition to yogurts, total polyphenolic content and antioxidant activity, as well as their stability along the fermentation process, the control and supplemented yogurt with the HWE were individually extracted twice with methanol/water/acetic acid solutions (80:19:1; v/v/v) along different fermentation times. The resulting extracts were concentrated, freeze-dried, and analyzed by the Folin–Ciocalteu protocol (µg GAE/100 g of yogurt fresh weight). For the antioxidant activity, the ABTS•^+^ method (µg Trolox equivalents/100 g of yogurt fresh weight) was selected given its simplicity. The same concentrations and solvents as those described for the HWE and pHWE extracts were used.

### 2.6. Anti-Inflammatory Potential

#### 2.6.1. Inhibition of Chemically-Induced NO• Production

The NO• scavenging method was adapted from Catarino et al. [21]. Briefly, 100 µL of the pHWE, solubilized in phosphate buffer at pH 7.4 at (260 µg/mL), was mixed with 100 µL of sodium nitroprusside 3.33 mM (also in buffer) and incubated under a fluorescent lamp (Tryun 26 W) for 15 min. Afterwards, 100 µL of Griess reagent (0.5% sulphanilamide and 0.05% naphthyletylenediamine dihydrochloride in 2.5% H_3_PO_4_) was added, and the mixture was incubated in the dark for 10 min. The absorbance was measured at 562 nm. NO• scavenging was expressed as % of inhibition.

#### 2.6.2. Inhibition of NO• Production in LPS-Stimulated Macrophages Cell Line Raw 264.7

The extracts were solubilized in sterile phosphate-buffered saline (PBS) with 2% (v/v) dimethyl sulfoxide (DMSO) and filtered through a cellulose acetate 0.22 µm sterile syringe filter (Frilabo, Maia, Portugal) under sterile conditions. The solutions were then diluted to achieve 281–1490 µg/mL of pHWE in the culture medium, with a final concentration of dimethyl sulfoxide (DMSO) lower than 0.1% (v/v). The medium was composed of Dulbecco’s Modified Eagle Medium (DMEM, A13169050, Applichem, Darmstadt, Germany) supplemented with 10% non-inactivated fetal bovine serum (Alfagene, Carcavelos, Portugal), 100 U/mL penicillin, 100 μg/mL streptomycin, and 17.95 mM sodium bicarbonate, all from Sigma, St. Louis, MO, USA.

Raw 264.7 cells, a mouse leukemic monocyte macrophage cell line from American Type Culture Collection (ATCC TIB-71), were plated (3 × 10^5^ cells/well) and allowed to stabilize for 12 h. Afterwards, the cell medium was replaced, and the cells were pre-incubated with 50 µL of pHWE or phosphate buffer with or without (control) 0.1% DMSO, for 1 h. Raw 264.7 cells were later activated with 1 μg/mL lipopolysaccharide (LPS from Escherichia coli, serotype 026:B6, Sigma Chemical Co., St. Louis, MO, USA) for 24 h. Cell viability was assessed using 3-(4,5-Dimethylthiazol-2-yl)-2,5-diphenyl tetrazolium bromide (MTT, Acros Organics, Geel, Belgium). The NO• production was determined by a colorimetric reaction with the Griess reagent, as previously reported by Búfalo et al. [22].

### 2.7. Nutritional Properties of the Yogurt

Given that apple pomace water extracts display high carbohydrate contents [23], the nutritional properties of the control and the HWE yogurts were evaluated by measuring the total sugar content and the amount of reducing sugars using the phenol-sulfuric method [24] and the 3,5-dinitrosalicylic acid (DNS) method [25]. The results were expressed as lactose equivalents/100 g, as lactose is the main carbohydrate found in dairy products. To complete the data set, moisture and protein contents were determined, as previously described. The protein conversion factor (6.15) estimated for dairy products was used for protein quantification [14]. The ash content was assessed by determining the final residue after incineration at 500 °C for 3 h. Fat was calculated by difference. The energetic value was calculated (Equation (3)) according to the energetic parameter published by the European Parliament [26]. As water extracts are known to present polysaccharides, their energetic contribution (2 kcal/g) was also included, assuming that polysaccharides are not affected and are not consumed by lactic acid bacteria during fermentation:

Energy (kcal) = 4 × (g of protein + g of reducing sugars) + 9 × (g of lipids) + 2 × (g of added apple pomace aqueous extract polysaccharides)(3)

### 2.8. Statistical Analysis

All experiments were performed with at least three independent assays being represented as mean ± standard error of the mean. Data were statistically analyzed by a trial version of GraphPad Prism 6.01 software (OriginLab Corporation, Northampton, MA, USA) by one-way analysis of variance (ANOVA) followed by Tukey’s multiple comparison test. A factor of 0.001 was used, unless otherwise stated.

## 3. Results

### 3.1. Apple Pomace Extracts

The industrial apple pomace had a high water-content (81%), rendering high perishability to this by-product. Protein comprised 50 g/kg of its dry weight, while carbohydrates were the major components (720 g/kg dry weight basis) (Table 1). These included the 180 g/kg of free sugars, mainly Fru (77 mol %), and 530 g/kg of polysaccharides with Glc (41 mol %), GalA (19 mol %), Ara (12 mol %), Xyl (10 mol %), and Gal (9 mol %) being the main sugars. This composition was characteristic of apple polysaccharides and reflects the presence of pectic polysaccharides as soluble dietary fiber and hemicelluloses and cellulose as insoluble dietary fiber [27,28]. Alongside these carbohydrates, polyphenols were also present. Therefore, hot water extraction was performed as it represents a cheap, non-toxic, environmentally friendly extraction procedure and is easily implementable at an industrial scale [5], in contrast to extractions using common organic solvents. To prevent polyphenol oxidation, diluted acetic acid was used [29].

HWE represented 29% of dry apple pomace and presented 11 g/kg of polyphenols (Table 1). This resulted from the co-extraction of carbohydrates (950 g/kg) and protein (13 g/kg), which accounted for 39% and 7% of that initially present in the apple pomace (Figure 1 and Table 1). The mass balance indicated that about 3.26 g GAE of polyphenols per kg of apple pomace were extracted. These yields were higher than those obtained with water at room temperature (2.6 g/kg of apple pomace) [30], and lower when using methanol (3.6 g/kg of apple pomace) or acetone (6.48 g GAE/kg of apple pomace) [9]. It is known that polysaccharides may interact with polyphenols, impairing their transfer from the fruit to the water fraction [31]. However, when using methanol and acetone, solvents that are of chaotropic nature, these interactions are disrupted and polyphenols become soluble [32], explaining the higher yields for methanol and acetone.

In order to infer about possible polyphenols–polysaccharides interactions in the HWE, ultrafiltration was performed using a 10 kDa ultrafiltration membrane. This process is based on the principle that apple polyphenols present, on average, a degree of polymerization of 5 [33], they would diffuse along the membrane unless being retained by any interaction phenomenon. According to the data presented in Table 1, 11% of the polyphenols from the HWE remained in the high molecular weight fraction. This fraction accounted for 6.9% of the apple pomace and was highly rich in polysaccharides (777 g/kg), mostly composed of Ara (35 mol %), GalA (36 mol %), and Glc (9 mol %). Ara and GalA are carbohydrates characteristic of pectic polysaccharides [28] while Glc is generally associated to glucans [23]. Therefore, it is possible to infer that the polyphenols present in the high molecular weight fraction were retained as a result of interactions with pectic polysaccharides and glucan fractions, as reported to occur between wine polyphenols and polysaccharides [34,35]. However, it is feasible that this retention mostly occurred as a result of covalent interactions between polyphenols and polysaccharides, due to the reactions between polyphenol quinones, formed by oxidation reactions and nucleophilic compounds of the cell wall [29]. In such case, polyphenols may establish bridges between different polysaccharide structures yielding chimeric structures [7]. Therefore, it is also possible to infer that some of the polyphenols present in the low molecular weight material (75% w/w of the HWE) were probably covalently associated to carbohydrates. Sugar analysis of this fraction showed the prevalence of GalA (45 mol %) and Ara (15 mol %), thus suggesting that they were associated to pectic oligosaccharides that globally represented 250 g/kg of the low molecular weight material. Such complexes are also present in the final extraction residue where polysaccharides represented 595 g/kg. Given the detection of GalA (13 mol%), Ara (8 mol%) and Gal (8 mol%), Glc (51 mol%), and Xyl (14 mol%), it is feasible that in addition to pectic polysaccharides, polyphenols were covalently linked to glucans [28,36].

To better understand the nature of the polyphenols and their bioactivity, the HWE was subjected to solid-phase extraction based on the principle that polyphenols, which have hydrophobic character, would be retained on the C18 cartridge while carbohydrates, which are hydrophilic molecules, would elute from the cartridge. The hydrophobic fraction, named pHWE, corresponded to 1.6% of the dry apple pomace. According to Folin–Ciocalteu’s method, polyphenols represent 149 g/kg and accounted for 63% of those in the HWE. The remaining 37% of the polyphenols eluted from the cartridge alongside with the HWE carbohydrates. These probably have the contribution of free sugars, which are known to have a reducing capacity and therefore interfere in the Folin–Ciocalteu method. However, the hypothesis that polyphenolic structures exist in this fraction as a result of covalent bonding to polysaccharides should not be discarded.

UHPLC-DAD-ESI-MS^n^ analysis showed that the pHWE was mainly composed of flavonols (Table 2) which included quercetin-3-*O*-galactoside (27%), quercetin-3-*O*-rhamnoside (23%), quercetin 3-*O*-arabinofuranoside (13%), and the dihydrochalcone phloretin-2-*O*-glucoside (14%). Quercetin-3-*O*-xylanoside (8%), quercetin-3-*O*-glucoside (7%), quercetin-*O*-pentoside (3%), quercetin-3-*O*-rutinoside (3%), and quercetin 3-*O*-arabinopyranoside (2%) were also detected as minor compounds, which was in agreement with previously reported work concerning the polyphenolic composition of apple pomace [5,37]. However, this analysis only explained 77% (115 mg/g of extract) of the polyphenols detected by the Folin–Ciocalteu method (149 mg/g of extract). According to Millet et al. [38], this difference, together with the fact that only 11% of the extract composition was explained, is highly suggestive of the occurrence of polyphenol oxidation products formed during apple processing [39]. Oxidation products of polyphenols are formed in very low amounts and present newly formed linkages [40], hardly quantitative by common ultra-high-pressure liquid chromatography (UHPLC) techniques [41]. These include hydroxycinnamic acid, dihydrochlacones, and flavan-3-ols oxidations products, already shown to be present in apple pomace [8].

### 3.2. Antioxidant and Anti-Inflammatory Potential

To provide evidence of the antioxidant potential of aqueous extracts from apple pomace, two widespread chemical models, the DPPH• and ABTS•^+^ radical inhibition assays, were used. As represented in Table 3, the EC_50_ values of the HWE for the DPPH• and ABTS•^+^ methods were 1.34 and 0.53 mg/mL, respectively. The increment of polyphenols in the purified fraction (pHWE) was reflected on the extract’s antioxidant activity, which exhibited DPPH• and ABTS•^+^ EC_50_ of 82.4 and 35.2 µg/mL, respectively (Table 3). When expressing the antioxidant activity with reference to total polyphenols (Table 3), it was observed that pHWE accounted for 92% of the HWE antioxidant activity. These results suggested that most of the compounds responsible for the antioxidant properties of the HWE were recovered after purification using C18 cartridges. The pHWE also presented the capability to inhibit the formation of OH• (6.75 mannitol equivalents/g of extract) generated by Fenton reactions from a ferric-ascorbate-EDTA-H_2_O_2_ system [20]. This can be attributed to both iron chelation and direct scavenging of OH• by polyphenols. However, in contrast to DPPH• and ABTS•^+^ which are not biological radicals, OH• is present in biological systems, resulting from Fenton reactions and cellular processes such as cell respiration and inflammation, prone to damage cellular lipids, proteins, and nucleic acids [42]. Closer extrapolations to in vivo effects could be inferred when complementing with other radical generating systems, either using enzymes such as xanthine/xanthine oxidase or cellular models such as activated neutrophils [43].

Nitric oxide (NO•) has been recognized as a versatile player in several biological mechanisms, including endothelial cell function and inflammation, turning it into a biomarker in the screening of new anti-hypertensive and anti-inflammatory drugs [44]. Therefore, the potential capability of the aqueous extracts from apple pomace to regulate NO•-driven processes was inferred through its ability to scavenge chemically generated NO•. As represented in Table 3, at a concentration of 130 µg/mL, the pHWE inhibited 35% of the NO• chemically generated, which is in accordance with the antioxidant properties previously described in this work. This inhibition was, however, lower than that produced by ascorbic acid (57%).

**Figure 2:**
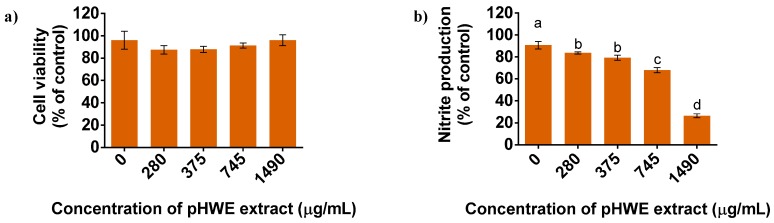
Treatment of mouse macrophage cell line, Raw 264.7, with apple pomace extracts, followed by incubation with lipopolysaccharide (LPS) from *Escherichia coli* as in vitro model of inflammation. (**a**) Cell viability (% of the control) of Raw 264.7 cells after incubation with polyphenolic-rich hot water extract (pHWE) at 0–1490 μg/mL in phosphate buffer/dimethyl sulfoxide (DMSO) (99.9:0.01; v/v). (**b**) Inhibitory effect of pHWE on LPS-induced nitrite production (% of the control) in Raw 264.7 cells. Data represent mean ± standard deviation of 3 independent assays. Different letters indicate statistical significance between pHWE concentrations (a,b,c, and d, *p* < 0.001) compared to control by one-way ANOVA followed by Tukey’s Multiple comparison test.

To provide closer evidence of anti-inflammatory effects occurring in vivo, which could potentiate the valuation of apple pomace for the development of functional foods, the pHWE was tested on LPS-stimulated Raw 264.7 cells, by measuring their effect on the accumulation of nitrites in the culture medium, an indicator of NO^•^ production. Indeed, macrophages activated by the Toll-like receptor 4 (TLR4) agonist LPS, produce large amounts of NO• and constitute a well-described in vitro model of inflammation, useful for the screening of molecules with anti-inflammatory activity [44]. In a first approach, the occurrence of possible cytotoxic effects triggered by the pHWE was evaluated by determining the cellular viability of Raw 264.7 macrophages, stimulated with LPS (Figure 2a). The presence of 0.1% DMSO did not affect the cell viability which was similar (*p* > 0.05) to the control in all tested concentrations. Accordingly, previously reported data showed that pure quercetin-glycosylated derivatives and phloridzin are not cytotoxic for the corresponding concentrations herein tested, as evaluated in similar cell models [45,46].

The anti-inflammatory potential of the pHWE was measured by the reduction of nitrite accumulated in the culture medium in comparison with the amount released by untreated LPS-stimulated Raw 264.7 cells. When treated with LPS, NO• released into the culture medium by macrophages increased from the basal value of 0.2 µM to 22 µM. Yet, when pre-incubated with pHWE in all non-cytotoxic concentrations (280, 375, 745, and 1490 μg/mL), the NO• released by macrophages was limited to 26%–84% of the value observed for non-treated cells (Figure 2b) in a concentration-dependent manner.

Some of the major polyphenols present in the apple pomace aqueous extracts have been previously reported to possess anti-inflammatory properties. For example, quercetin, a flavonol, and some of its glycosylated derivatives were shown to inhibit NO• production in LPS-induced Raw 264.7 cells and to modulate several inflammatory signaling cascades [31,33]. Phloridzin, a dihydrochalchone, has also been described to modulate inflammatory responses [32]. Nevertheless, direct relation of the observed anti-inflammatory effect with the presence of polyphenols is still not possible to establish, since despite pHWE purification, other unknown compounds representing 85% of the extract, possibly apple polyphenol oxidation products formed during processing [29], may also be responsible for the effects. Furthermore, no comparisons with the HWE could be established given its poor solubility in phosphate-buffered saline with 2% (v/v) dimethyl sulfoxide (DMSO) at room temperature and the adverse effects on the viability of Raw 264.7 cells when using higher concentrations of DMSO. Although a deeper consolidation through the measurement of pro- and anti-inflammatory interleukins and/or other anti-inflammatory markers is required, these results evidence the possible valuation of apple pomace as a potential anti-inflammatory nutraceutical.

### 3.3. Application as a Food Ingredient in Yogurt Formulation

**Figure 3:**
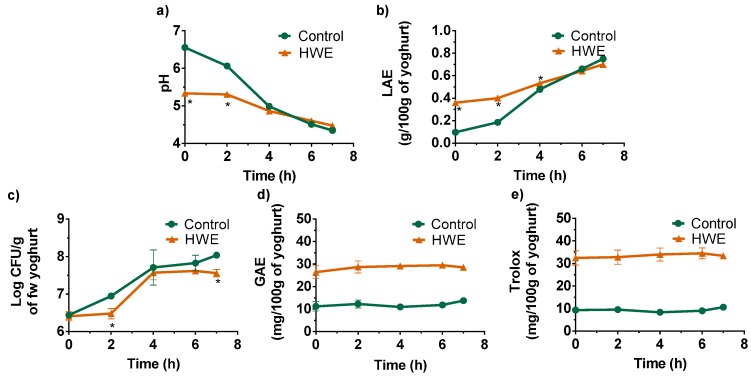
Evolution of (**a**) pH, (**b**) titratable acidity (expressed as lactic acid equivalents (LAE)/100 g of fresh weight yogurt)), (**c**) Streptococcus thermophilus counts (Log CFU/g of fresh weight yogurt), (**d**) total polyphenolic content (mg gallic acid equivalents (GAE)/100 g of fresh weight yogurt), and (**e**) antioxidant activity (mg of Trolox/100 g of fresh weight yogurt) along the fermentation process for the control and supplemented yogurt with the HWE.

Given the presence of polysaccharide/polyphenol complexes and the possible antioxidant and anti-inflammatory properties that could be attributed to apple pomace extracts, their potential to be used as ingredients for food formulations was tested. Yoghurt was chosen as it is a worldwide, ready-to-eat product with a high nutritional value and positive bioactive effects that can be reinforced by the addition of other components [47,48,49]. As a result, HWE was incorporated into yogurt formulations to reach 3.3% (w_extract_/w_milk_). The incorporation of the HWE resulted in a mixture with an initial pH of 5.34, 19% lower than the control (6.56), which is in accordance with the higher titratable acidity observed (0.361 versus 0.097 g lactic acid/100 g of yogurt) (Figure 3a,b). This effect was similarly observed when adding wine grape pomace to yogurts [47] and could be related to naturally occurring organic acids, such as malic acid, in apple pomace. The similar number of *Streptococcus thermophilus* counts (6.4 Log CFU/g of yogurt) observed in both yogurts (Figure 3c) is indicative that the viability of lactic acid bacteria is not affected by the supplementation. As represented in Figure 3d, the total polyphenolic content of the control yogurt (11 ± 2.0 mg GAE/100 g of fresh weight yogurt), whose activity can also be attributed to Tyr, Trp and Phe [48], more than doubled with the addition of the extract (29 ± 3.0 mg GAE/100 g of fresh weight yogurt). This represented a higher phenolic content compared to the use of hazelnut skins [49], but inferior when supplementing yogurts with wine grape pomace [47]. These results are attributed to differences at quantitative and qualitative levels of the phenolic structures present in the various agro-industrial by-products. When compared to the total polyphenols added to the yogurt mixture (32.1 mg GAE) with the amount determined in the yogurt mixture after control subtraction, it was observed that only 56% were determined by the Folin–Ciocalteu method, similar to what was observed when protein was added to polyphenols [50]. This variation is possibly attributed to the capability of apple pomace polyphenols to interact with milk proteins, thereby blocking the polyphenolic aromatic rings responsible for their antioxidant properties. Nevertheless, an increase of the antioxidant activity in more than three-fold (from 9 ± 1 to 32 ± 4 mg trolox/100 g of fresh weight yogurt) was measured by the ABTS•^+^ method (Figure 1e), reflecting the antioxidant properties described for the HWE.

As represented in Figure 3a–c the pH decrease, concomitant with the increase of the titratable acidity and *S. thermophilus* counts, demonstrated that the mixtures with and without apple pomace HWE were fermented, yielding a control and a supplemented yogurt. Nevertheless, *S. thermophilus* in the HWE yogurt appeared to have an increased lag phase and reached lower counts at the end of fermentation when compared to the control, which might indicate that the tested concentration may have an inhibitory effect on their growth. Although lactic acid bacteria are generally isolated and remain viable in acidic foods, their growth is inhibited at low pH levels, in particular the growth of *S. thermophilus* [51]. Therefore, it is possible that the extended lag phase observed in the HWE yogurt resulted from its initial acidic pH when compared to the one observed for the control. Nevertheless, the *S. thermophilus* counts exceeded the 7 Log CFU/g, with a final pH below 4.5, which are positive critical factors to inhibit pathogenic microorganisms such as *Listeria monocytogenes*, *Salmonella*, or *Escherichia coli*, and to assure the stability of the product [52,53]. Furthermore, it was observed that the yogurt total polyphenolic content and antioxidant activity (Figure 3d,e) remained unchanged during the fermentation process. This suggested that the polyphenols incorporated into yogurt were not affected. However, given the capability of bacteria to metabolize polyphenols, it is feasible that some apple pomace polyphenolic structures were converted to metabolites that still possess antioxidant properties. These changes might not be reflected on the overall total phenolic content and antioxidant activity, as measured by the Folin–Ciocalteu and ABTS•^+^ methods. Reports on yogurts supplemented with hazelnut skins [49] and grape pomace [47] suggest that their antioxidant properties can be relatively stable for at least two weeks.

Table 4 shows the nutritional composition, expressed in g/100 g of fresh weight yogurt, and the energetic value, expressed in kcal/100 g of fresh weight yogurt of the control and supplemented yogurt. The nutritional analysis revealed that water was the major component (87%–89%) in both formulations. The addition of the HWE resulted in an increase of the yogurts total sugars from 5.5% to 7.3% and of the reducing sugars from 4.5% to 5.1%. As lactic acid bacteria, particularly *S. thermophilus*, preferentially use lactose over glucose [54], these increments can be attributed to some glucose and fructose and to oligosaccharides and polysaccharides from the HWE that remain after fermentation. From the latter, two distinct groups could be highlighted: (1) high molecular weight (>10 kDa) polysaccharide/polyphenol complexes to which higher short-chain fatty acid production and polyphenol-derived metabolites are attributed than to polyphenols and polysaccharides alone [55], and (2) pectic oligosaccharides to which prebiotic properties are also attributed [56]. It has been shown that these carbohydrate structures also improve yogurt firmness [57]. Protein, fat, and ash corresponded to about 3.2%, 1.5%, and 0.9%, respectively. Overall, the energetic values of both yogurts were 47 and 49 kcal/100 g of fresh weight for the control and HWE yogurt, respectively.

## 4. Conclusions

In this work, it was shown that by hot water extraction under acidic conditions more than 3 g/kg of dry apple pomace could be obtained. Ultrafiltration demonstrated that in addition to apple native polyphenols, some phenolic structures were probably attached to the high molecular weight material (>10 kDa). Separation of HWE polyphenols by solid-phase extraction allowed us to infer the potential antioxidant and anti-inflammatory capacities, as shown by their scavenging ability towards NO• in chemical and cellular models. When applied to yogurt formulations, apple pomace HWE allowed for achieving a final product with improved fiber content and antioxidant properties when compared to the plain yogurt. However, as this study involves only in vitro assays, its extrapolation to humans cannot be done. This results from the fact that DPPH• and ABTS•^+^ radicals, although suggestive of antioxidant properties, do not represent physiological radicals while the OH• and NO• scavenging assays were performed using in vitro models. Therefore, these results should be considered as a proof of concept for food chemists and industrials. For the evaluation of possible health benefits, in vivo studies addressing the bioavailability of the polyphenols and the dosage required to observe any antioxidant or anti-inflammatory effects are necessary. In fact, these requirements are regulated by the European Food Safety Authority (EFSA) and must meet not only the requirements for health claims (Regulation (EC) No 1924/2006 of the European Parliament and of the Council), but also for safety (Regulation (EU) No 1169/2011 of the European Parliament and of the Council of 25 October 2011) before any commercialization of a food product stating health benefits. In this context, no statement of the type “antioxidant and anti-inflammatory apple pomace extract/yoghurt” cannot be used at this stage and any commercial exploitation of the developed yoghurt formulation must naturally assure its safety.

## Figures and Tables

**Table 1:** Yield (%), monosaccharide (molar%), carbohydrate (%), protein (%), and polyphenolic composition (g gallic acid equivalents (GAE)/kg) of industrial apple pomace, hot water extract (HWE), high molecular weight material (HMWM), low molecular weight material (LMWM), and extraction residue.

Fractions	Yield (%)	Yield of Carbohydrate (%)		Carbohydrate (mol%)									Total Carbohydrate (%)		Total Protein (%)	Total PC (g GAE/kg)
				Rha	Fuc	Ara	Xyl	Man	Gal	Glc	Fru	GalA				
Apple pomace			Polysaccharides	1	1	12	10	5	9	41		19	53.4	71.7	5.2	ND
			Free Sugars							23	77		18.3			
HWE	29.6	39.2	Polysaccharides	3	1	25	3		10	9		50	42.9	94.9	1.3	11
			Free Sugars							18	82		52.0			
HMWM	6.9	7.5	Polysaccharides	1	t	35	6	1	10	9		36	77.7	77.7	ND	5
LMWM	22.3	27.7	Polysaccharides	1	t	15	2		6	34		42	33.5	89.0	ND	9
			Free Sugars							6	94		55.5			
Residue	67.4	55.9	Polysaccharides	1	1	8	14	4	8	51		13	59.5	59.5	7.1	

t = trace; ND = not determined.

**Table 2:** Retention time (RT), mass spectrum (MS), and polyphenolic composition (mg/g of extract) of pHWE.

N°	RT	Compound	λ_max_	MS (*m*/*z*)	MS^2^ (*m*/*z*)	Extract
						pHWE
**1**	12.3	Quercetin-3-*O*-rutinoside ^a^	254, 353	609	463, 301	3.27 ± 0.06
**2**	12.6	Quercetin-3-*O*-galactoside ^b^	256, 354	463	301	31.37 ± 0.32
**3**	12.7	Quercetin-3-*O*-glucoside ^a^	256, 353	463	301	8.45 ± 0.10
**4**	13.2	Quercetin-3-*O*-xylanoside ^b^	256, 354	433	301	8.88 ± 0.10
**5**	13.4	Quercetin 3-*O*-arabinopyranoside ^b^	243, 352	433	301	2.31 ± 0.04
**6**	13.5	Quercetin 3-*O*-arabinofuranoside ^b^	256, 352	433	301	15.09 ± 0.16
**7**	13.7	Quercetin-*O*-pentoside ^b^	256, 351	433	301	3.32 ± 0.05
**8**	13.8	Quercetin-3-*O*-rhamnoside ^b^	256, 350	447	301	26.05 ± 0.27
**9**	14.9	Phloretin-2-*O*-glucoside ^a^	227, 284	435	273	15.96 ± 0.20
					**Total**	114.75 ± 1.25

Identification was performed based on (a) the corresponding standard; (b) UV and MS^n^ spectra, plus elution order previously described in the literature [5,37].

**Table 3:** Total polyphenolic content (TPC) and antioxidant (DPPH•, ABTS•^+^, OH•, NO•) activity of the hot water extract before (HWE) and after the purification (pHWE).

Extract	TPC	DPPH•	ABTS•^+^	OH•	NO•
**HWE**	10.7 ± 0.2	1339 ± 211 (14.2 ± 1.7)	532 ± 11.5 (5.69 ± 0.12)	-	-
**pHWE**	149 ± 1.87	82.4 ± 11.2 (12.3 ± 1.7)	35.2 ± 5.9 (5.23 ± 0.44)	6.75 ± 0.45	35.2 ± 5.9
**AA**	-	2.70 ± 0.30	2.68 ± 0.03	-	57.3 ± 2.3

The first and second values for the DPPH• and ABTS•^+^ are expressed in terms of EC_50_ (µg of extract/mL) and as relative antioxidant capacity with reference to total polyphenols (µg GAE of extract/mL), respectively. OH• scavenging was expressed as mannitol equivalents (mmol/g of extract), and for the NO• method as percentage of inhibition at 130 µg/mL. Values are compared to ascorbic acid (AA).

**Table 4:** Nutritional composition expressed as g/100 g of fresh weight control and supplemented yogurt with apple pomace HWE.

Components	Control	HWE
**Moisture**	88.8 ± 0.0	87.2 ± 0.1
**Total Sugars**	5.45 ± 0.12	7.30 ± 0.18
**Reducing Sugars**	4.49 ± 0.10	5.05 ± 0.04
HWE polysaccharides *		1.43 ± 0.01
**Protein**	3.32 ± 0.04	3.21 ± 0.08
**Fat**	1.68 ± 0.15	1.46 ± 0.07
**Ash**	0.75 ± 0.01	0.84 ± 0.01
**Energetic (kcal)**	46.5 ± 1.1	49.1 ± 0.7

* Assuming that HWE polysaccharides are preserved during fermentation.
